# Cone-beam CT findings during prostate artery embolization for benign prostatic hyperplasia-induced lower urinary tract symptoms: a case report

**DOI:** 10.1186/s12894-017-0311-6

**Published:** 2017-12-19

**Authors:** Chia-Bang Chen, Chen-Te Chou, Yao-Li Chen

**Affiliations:** 1 Department of Radiology, Changhua Christian Hospital, No. 135, Nan-Hsiao Street, Changhua, 500 Taiwan; 2 Department of Biomedical Imaging and Radiological Science, National Yang-Ming Medical University, No.155, Sec. 2, Linong Street, Taipei, 112 Taiwan; 3 Transplant Medicine and Surgery Research Centre, Changhua Christian Hospital, No. 135, Nan-Hsiao Street, Changhua, 500 Taiwan; 4 School of Medicine, Chung Shan Medical University, Taichung City, 40201 Taiwan; 5 School of Medicine, Kaohsiung Medical University, Kaohsiung, Taiwan; 6 Department of General Surgery, Changhua Christian Hospital, No. 135, Nan-Hsiao Street, Changhua, 5006 Taiwan

**Keywords:** Cone-beam computed tomography, Prostatic artery embolization, Benign prostatic hyperplasia, Lower urinary tract symptoms

## Abstract

**Background:**

Cone-beam computed tomography (CBCT) is a new and useful technique for angiographic procedures. Prostatic artery embolization (PAE) has emerged as a promising treatment modality for lower urinary tract symptoms (LUTS) caused by benign prostatic hyperplasia (BPH). CBCT can be helpful for PAE to determine the correct arteries for embolization, and to show any occlusion of these arteries.

**Case presentation:**

Herein, we report on a patient who underwent CBCT-guided PAE as treatment for BPH-induced LUTS, present the imaging findings, and provide technical suggestions.

**Conclusions:**

PAE is an effective, minimally invasive modality for the treatment of LUTS due to BPH, and contrast CBCT can help visualize and demonstrate occlusion of the prostatic arteries.

## Background

Benign prostatic hyperplasia (BPH) is a very common benign disease in elderly men, with a prevalence rate ranging from 50 to 90% [1]. Acute urinary retention and lower urinary tract symptoms (LUTS) are common complications of BPH, and many different treatment modalities are available. Surgical therapies are indicated for patients with severe LUTS or for non-responders to medical treatment. Transurethral resection of the prostate (TURP) is the most common surgical modality for patients with LUTS due to BPH; however, many patients develop complications after the operation. Prostatic artery embolization (PAE) is a minimally invasive technique that has been shown to be effective at relieving LUTS in patients with BPH. Cone-beam computed tomography (CBCT) is a new imaging technique for angiographic procedures. The modality provides high resolution three-dimensional and cross-sectional images. Administration of intravascular contrast medium results in good enhancement of the vessels and target lesion after which the CBCT images can be reconstructed into images that resemble angiographic images. Herein, we present the first case in Taiwan of BPH-related LUTS that was treated using CBCT-guided PAE. CBCT was used to visualize and show total occlusion of the prostatic arteries. The technical aspects of the procedure are also provided.

## Case presentation

An 85-year-old man presented with a two-year history of progressive urinary frequency, urgency, nocturia, and acute urinary retention. Sonography revealed an enlarged prostate gland measuring about 127 mL in volume. Based on the symptoms and sonographic findings, the diagnosis of BPH with LUTS was made, and a Foley catheter was inserted immediately to provide short-term relief of acute urinary retention. Surgical treatment rather than medical therapy was provided considering the time it would have taken for medical therapy to take effect and the patient’s wish to avoid having an indwelling Foley catheter for a prolonged period. PAE rather than TURP was attempted because of the patient’s advanced age. Before the procedure, the patient’s score on the symptom index of the international prostate symptom score (IPSS) was 21 and his score on the quality of life index was 4. Prostate gland volume (162 mL) was estimated on axial T2-weighted magnetic resonance (MR) images.

**Fig. 1:**
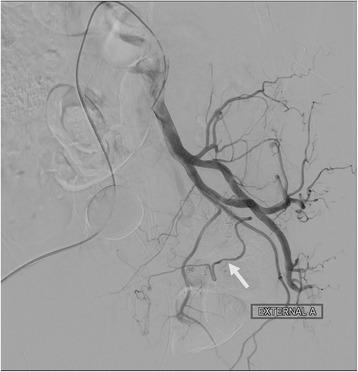
Angiogram of the left internal iliac artery shows the LPA (arrow) arising from the left internal pudendal artery

**Fig. 2:**
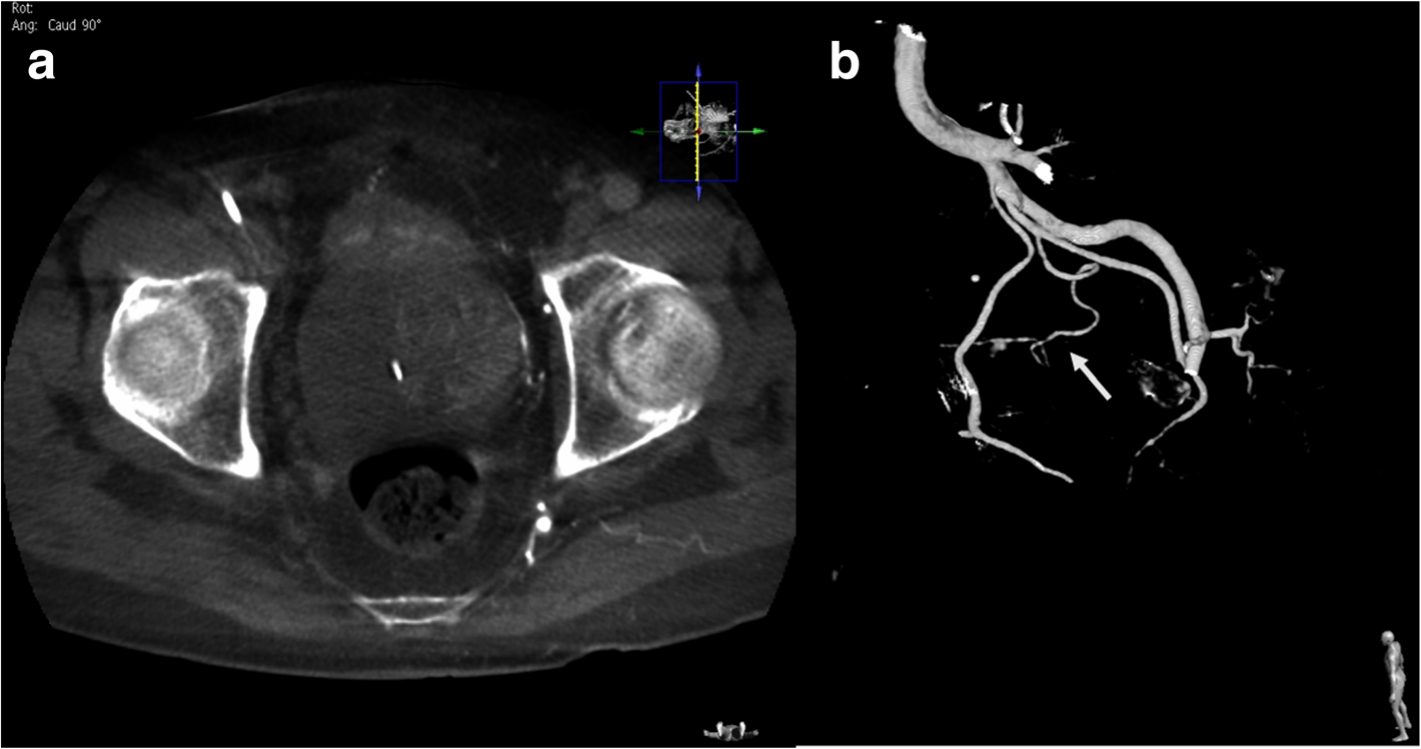
Contrast CBCT of the left internal iliac artery. **a** Good parenchymal enhancement of the left aspect of the prostate gland was visualized. **b** Three-dimensional reconstructed image demonstrates the vascular anatomy of the LPA (arrow)

**Fig. 3:**
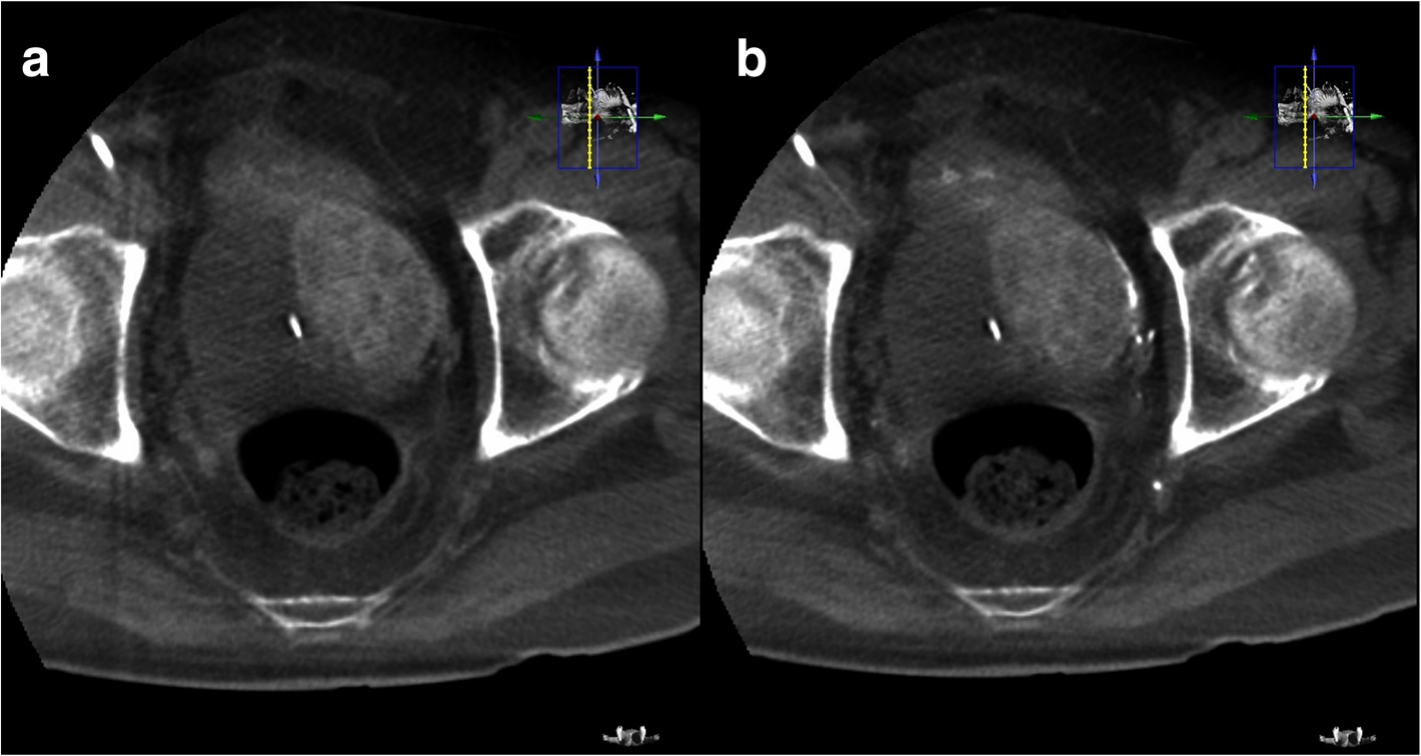
**a** Non-contrast CBCT after embolization of the LPA shows retention of contrast medium in the left aspect of the prostatic parenchyma without filling defect. Some contrast medium was excreted from the kidneys and accumulated in the urinary bladder. **b** Contrast CBCT after embolization of the LPA shows no further enhancement of the left aspect of the prostatic parenchyma, suggesting adequate embolization

Before the embolization procedure, 10 mL of diluted iodinated contrast medium (a mixture of 50% iodinated contrast medium plus 50% normal saline solution) was injected into the balloon of the Foley catheter to help localize the prostate gland under fluoroscopy. The left common and internal iliac arteries were accessed using a 4 Fr. J-curve catheter (Terumo, Tokyo, Japan) via the right femoral approach. Angiography of the left common and internal iliac arteries was performed, with 10 mL pure contrast medium injected by power injector at a rate of 2 mL/s, and with an image intensifier at a left anterior oblique (LAO) projection angle of 55 degrees to visualize the vascular anatomy (Fig. 1). A 1.98 Fr. coaxial microcatheter (Asahi INTECC CO, Nagoya, Japan) was used to access the left prostatic artery (LPA), which in our patient arose from the left pudendal artery. Contrast CBCT was performed from the left internal iliac artery, with 20 mL of a 67%:33% saline-to-contrast medium mixture injected by power injector at a flow rate of 1 mL/s and with an image delay of 6 s, and also from the superselected LPA with 10 mL of diluted contrast medium injected by power injector at a flow rate of 0.5 mL/s and with an image delay of 6 s, to provide a three-dimensional view of the detailed vascular anatomy and to visualize the parenchymal enhancement of the left aspect of the prostate gland without filling defect (Fig. 2). Embozene Color-Advanced Microspheres measuring 250 μm in diameter (Celonova Biosciences Inc., San Antonio, USA) were slowly delivered after dilution with 5 mL contrast medium, until near stasis was achieved. Non-contrast CBCT demonstrated retention of the contrast medium without filling defect, and contrast CBCT revealed no further enhancement of the left aspect of the prostate gland parenchyma (Fig. 3).

**Fig. 4:**
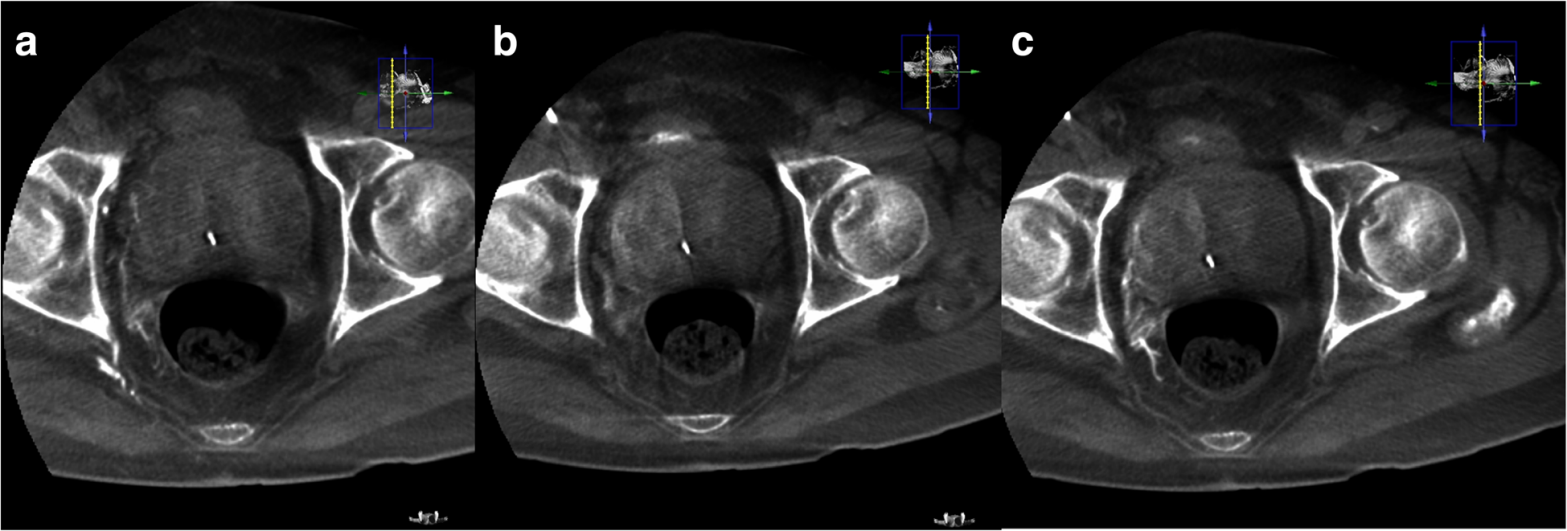
**a** Contrast CBCT of the RPA shows small distal branches supplying the right aspect of the rectum. Decreased contrast medium retention in the left aspect of the prostate gland can be seen, as compared with Fig. 3(a). **b** Non-contrast CBCT after embolization of the RPA shows retention of contrast medium in the right aspect of the prostatic parenchyma and the distal branches, without filling defect. **c** Contrast CBCT after embolization of the RPA shows no further enhancement of the right aspect of the prostate gland

A 5 Fr. RIM catheter (Cook Medical, Bloomington, Indiana) and a 1.98 Fr. coaxial microcatheter were used to access the right prostatic artery (RPA), which arose from the anterior division of the right internal iliac artery. Conventional angiography with a right anterior oblique (RAO) projection of 55 degrees was performed to demonstrate the vascular anatomy. Contrast CBCT was performed both from the right internal iliac artery and super selected RPA to demonstrate the vascular anatomy and to visualize parenchymal enhancement of the right aspect of the prostate gland without filling defect. In addition, decreased retention of contrast medium in the left aspect of the prostate gland parenchyma was depicted, as compared with the CBCT during embolization of the LPA. The contrast CBCT also clearly demonstrated that some small distal branches of the RPA supplied the right aspect of the rectum (Fig. 4). Embolization of these branches using microcoils could not be performed because they were too small to access. Embolization of the RPA was also performed with Embozene microspheres (250 μm) diluted with 5 mL contrast medium, and contrast CBCT was performed to ensure occlusion of the RPA, by visualizing retention of the contrast medium without filling defects and without further enhancement of the right aspect of the prostate gland parenchyma. Only 1 mL of the Embozene microspheres was delivered during this procedure.

There were no postprocedural complications. The patient was discharged the day after the procedure and the Foley catheter was removed 4 days after discharge. The patient reported a several-day history of passing mucus-containing stool after the procedure, but the side effect was self-limiting. His symptom index score on the IPSS decreased from 21 before the procedure to 5 about 3 weeks after the procedure and his quality of life index score decreased from 4 to 0. At the two-month follow-up, the prostate volume as measured on MR images was 76 mL.

## Discussion

In the previous literature, computed tomography angiography (CTA) was recommended before the embolization procedure to evaluate the pelvic vascular anatomy [2]. However, contrast CBCT has been shown to clearly demonstrate the vascular anatomy during the embolization procedure [3], and thus it is no longer necessary to perform CTA before the procedure. Furthermore, CBCT can demonstrate filling defects of the prostate gland due to accessory or collateral arterial supplies, and demonstrate occlusion of the prostatic arteries, by showing retention of contrast medium in the prostatic parenchyma without filling defects, and revealing no further parenchymal enhancement.

The contrast Foley balloon catheter can be used as a landmark of urinary bladder and prostate under fluoroscopic guidance [4], since the prostate is radiolucent. When accessing the prostatic arteries under fluoroscopy, the prostatic arteries can be located easily because they run just under the contrast Foley balloon, along a course to the midline of the pelvis. After successfully catheterizing the prostatic arteries, contrast CBCT can be used for confirmation, by demonstrating enhancement of the prostatic parenchyma, and also to detect arteries supplying adjacent organs to avoid non-target embolization.

In our patient, microspheres of 250 μm were used for embolization. After near stasis had been achieved, non-contrast CBCT showed retained contrast medium in the prostatic gland parenchyma without filling defect. However, decreased retention of the contrast medium in the left prostate gland parenchyma after embolization of the LPA was noted on contrast CBCT images, probably because much of the contrast medium had been flushed out by the blood flow from the RPA. This finding suggests that there are tiny collateral vessels connecting the LPA and RPA. Pisco et al. [5] reported that embolization of the bilateral prostatic arteries should be attempted because unilateral embolization results in a higher rate of treatment failure. In our patient, contrast CBCT revealed no further enhancement, and non-contrast CBCT showed no filling defects of retained contrast medium in the prostatic parenchyma, both indicating occlusion of the prostatic arteries. There were some small vessels branching from the RPA to the rectum and embolization of these branches using microspheres larger than 200 μm in diameter has been shown to be safe [6].

## Conclusions

In conclusion, PAE is an effective, minimally invasive modality for the treatment of LUTS due to BPH, and contrast CBCT can help visualize the prostatic arteries and detect accessory or collateral supplying vessels. Embolization of the prostatic arteries can be visualized using non-contrast and contrast CBCT by showing retention of contrast medium without filling defect and no further enhancement of the prostatic gland parenchyma. However, embolization in the bilateral prostatic arteries should be attempted to achieve a better clinical outcome.

## Abbreviation

BPH: benign prostatic hyperplasia
CBCT: cone-beam computed tomography
LUTS: lower urinary tract symptoms
PAE: prostate artery embolization
